# Recurrent 17q12 microduplications contribute to renal disease but not diabetes

**DOI:** 10.1136/jmg-2022-108615

**Published:** 2022-09-15

**Authors:** Stuart Cannon, Rhian Clissold, Kittiya Sukcharoen, Marcus Tuke, Gareth Hawkes, Robin N Beaumont, Andrew R Wood, Mark Gilchrist, Andrew T Hattersley, Richard A Oram, Kashyap Patel, Caroline Wright, Michael N Weedon

**Affiliations:** 1 Institute of Biomedical and Clinical Science, University of Exeter Medical School, Exeter, UK; 2 Exeter Kidney Unit, Royal Devon University Healthcare NHS Foundation Trust, Exeter, UK

**Keywords:** diabetes mellitus, endocrinology, nephrology

## Abstract

**Background:**

17q12 microdeletion and microduplication syndromes present as overlapping, multisystem disorders. We assessed the disease phenotypes of individuals with 17q12 CNV in a population-based cohort.

**Methods:**

We investigated 17q12 CNV using microarray data from 450 993 individuals in the UK Biobank and calculated disease status associations for diabetes, liver and renal function, neurological and psychiatric traits.

**Results:**

We identified 11 17q12 microdeletions and 106 microduplications. Microdeletions were strongly associated with diabetes (p=2×10^−7^) but microduplications were not. Estimated glomerular filtration rate (eGFR mL/min/1.73 m^2^) was consistently lower in individuals with microdeletions (p=3×10^−12^) and microduplications (p=6×10^−25^). Similarly, eGFR <60, including end-stage renal disease, was associated with microdeletions (p=2×10^−9^, p<0.003) and microduplications (p=1×10^−9^, p=0.009), respectively, highlighting sometimes substantially reduced renal function in each. Microduplications were associated with decreased fluid intelligence (p=3×10^−4^). SNP association analysis in the 17q12 region implicated changes to *HNF1B* as causing decreased eGFR (NC_000017.11:g.37741642T>G, rs12601991, p=4×10^−21^) and diabetes (NC_000017.11:g.37741165C>T, rs7501939, p=6×10^−17^). A second locus within the region was also associated with fluid intelligence (NC_000017.11:g.36593168T>C, rs1005552, p=6×10^−9^) and decreased eGFR (NC_000017.11:g.36558947T>C, rs12150665, p=4×10^–15^).

**Conclusion:**

We demonstrate 17q12 microdeletions but not microduplications are associated with diabetes in a population-based cohort, likely caused by *HNF1B* haploinsufficiency. We show that both 17q12 microdeletions and microduplications are associated with renal disease, and multiple genes within the region likely contribute to renal and neurocognitive phenotypes.

**WHAT IS ALREADY KNOWN ON THIS TOPIC**

- 17q12 microdeletions (OMIM: 614527) and microduplications (OMIM: 614526) are known to cause complex, multisystem syndromes, including diabetes and renal disease.
- Disease phenotypes in microdeletions are predominantly attributed to the haploinsufficiency of the 17q12 gene, *HNF1B*.

**WHAT THIS STUDY ADDS**

- Here we show that 17q12 microduplications as well as microdeletions contribute to renal disease in a population cohort.
- We also show that genes other than *HNF1B* in the 17q12 region are implicated in renal disease caused by microduplications.

**HOW THIS STUDY MIGHT AFFECT RESEARCH, PRACTICE OR POLICY**

- This study highlights the need to monitor duplication patients for renal disease, but also how further research to delineate the genes driving the complex syndromes is required.

## Introduction

Recurrent microdeletions (OMIM: 614527) and microduplications (OMIM: 614526) of ~1.4 Mb region of 17q12 have each been associated with a range of conditions including diabetes, renal disease and intellectual disability (see Rasmussen *et al* 1 for review). Despite incomplete penetrance and variable expressivity,^2^ some phenotypes present more consistently with microdeletions and others with microduplications.^3–5^ The microdeletion syndrome can include developmental kidney disease, early-onset diabetes mellitus, pancreatic hypoplasia, genital tract malformations, abnormal liver function tests, and neurodevelopmental disorders such as autism spectrum disorder and attention deficit hyperactivity disorder.^2 6^ The 17q12 microduplication is associated with a more variable phenotype that can include cognitive impairment, speech and motor developmental delay, brain anomalies, dysmorphic facial features, behavioural abnormalities, oesophageal atresia, renal anomalies and epilepsy.^3 7–9^ Renal function in 17q12 microduplications has not been characterised in a population cohort.

The 17q12 CNV region encompasses 15 protein-coding genes, including *HNF1B*, bounded by segmental microduplications. The association of the 17q12 deletion with diabetes and decreased kidney function is known to be caused by haploinsufficiency of *HNF1B*.^10 11^ Protein-truncating variants (PTVs) in *HNF1B* cause a similar phenotype^6 12 13^; however, *HNF1B* PTVs are not associated with neurodevelopmental disorders.^14^ It is unknown whether *HNF1B* triplosensitivity contributes to the phenotypes observed in individuals with 17q12 microduplications or whether other genes in the region also play a role in the deletion or microduplication phenotypes.

Individuals with 17q12 microdeletions and microduplications are often identified through clinical referral and present with distinct phenotypes.^15^ However, large data sets are required to determine the functional associations of rare CNVs with variable penetrance and expressivity in the population.^16^ The phenotypes of individuals with these variants when identified incidentally from the population have not been studied in depth. The UK Biobank (UKB), a population-based cohort of ~500 000 individuals, offers a unique opportunity to characterise the 17q12 locus in a population setting and to investigate the differences between deletion and microduplication phenotypes. In this study we assessed diabetes plus renal, liver and neurological phenotypes of individuals in the UKB with recurrent 17q12 microdeletions and microduplications. We demonstrate that microduplications are a cause of renal disease but not diabetes and provide evidence that genes other than *HNF1B* are driving both decreased fluid intelligence and renal function.

## Methods

### Study subjects

Data from 450 993 participants of European ancestry from the UKB were analysed in this study. The UKB cohort is described in detail elsewhere.^17 18^ Phenotypes were derived using International Classification of Diease codes (ICD9 and ICD10) as well as from Hospital Episode Statistics (HES) data, serum and urinary biomarkers, and UKB-defined traits such as bipolar and major depression status or fluid intelligence. All phenotypes are detailed in online supplemental table 1. Participants withdrawn prior to the time of submission were excluded from analysis.

10.1136/jmg-2022-108615.supp1 Supplementary data

### CNV calling

CNVs overlapping the 17q12 region were detected as outlined in Tuke *et al*.^19^ Briefly, SNP microarray data in the UKB were used to call CNVs using PennCNV V.1.0.4, with log R ratio (LRR) and B-allele frequency (BAF) values for 805 426 genome-wide probe sets provided by the UKB. All CNV calls were manually curated by inspecting plotted LRR and BAF. Large chromosomal aneuploidies and those with suspected mosaicism were excluded.

### SNP association analysis

SNP genotypes were generated from the Affymetrix Axiom UKB array (∼450 000 individuals) and the UK Biobank Lung Exome Variant Evaluation (UK BiLEVE) consortium array (∼50 000 individuals) in 106 batches of ∼4700 samples. This data set underwent extensive central quality control.^18^ High-quality imputed SNPs in the 17q12 region chr17:31827018–37956253 (GRCh37) (n=195 736) were extracted and then only those with minor allele frequency (MAF) >0.001 between the segmental microduplication regions (chr17:34442621–36711256) were included (n=535).

### Exome sequencing

Variants detected using exome sequencing of 184 532 UKB participants were annotated using the Ensembl Variant Effect Predictor^20^ with the LOFTEE plugin.^21^ Rare variants were included if they had a minor allele count (MAC) ≤30, LOFTEE high-confidence loss of function (LoF) or had a Combined Annotation Dependent Depletion (CADD) score >30.^22^ The aligned sequence data for all variants meeting these criteria were visually inspected using the Integrative Genomics Viewer 23 (IGV) to remove likely false positives.

### Phenotypes

#### Diabetes

Diabetes was defined as being one or more of the following: self-reported by participants or having an ICD9/ICD10 code for diabetes, or being on a diabetes treatment, or having glycated haemoglobin (HbA1c) ≥48 mmol/mol before recruitment.^24 25^

#### Renal and liver disease

Estimated glomerular filtration rate (eGFR mL/min/1.73 m^2^) was calculated using the Chronic Kidney Disease Epidemiology Collaboration Creatinine-Cystatin Equation 2012 and was used to classify end-stage renal disease (ESRD), as well as broader chronic kidney disease categories: eGFR ≥60, eGFR <60 and eGFR <45. ESRD and eGFR <45 classifications included individuals who had renal replacement therapies such as kidney transplant or dialysis. Phenotypes for structural malformations of the kidney and ureter as well as any other structural malformation encoded in the HES data were also included in the analyses. A continuous measure of albumin to creatinine ratio (ACR) was derived from urinary microalbumin and creatinine. Individuals with an undetectable level of microalbumin were included as the minimum value in the UKB (6.7 mg/L), as has been done previously.^26^ Serum biomarkers alkaline phosphatase (ALP), alanine aminotransferase (ALT), aspartate aminotransferase (AST), gamma glutamyl transferase (GGT), total bilirubin and direct bilirubin were used.

#### Fluid intelligence, general measures of functioning and neurodevelopmental/psychiatric disorders

Fluid intelligence, income, job class, educational qualification attainment level (qualification) and number of years in education were recorded. Fluid intelligence is measured as the number of questions (n=13) answered by participants in 2 min. Participants with diagnosed neurological or psychiatric disorders, such as intellectual disability or depression, were identified using HES codes. The developmental delay category combined neurodevelopmental disorders, bipolar, schizophrenia, depression, pervasive disorders, intellectual disability, epilepsy as well as structural malformations.

All phenotypes and associated codes are detailed in online supplemental table 1.

### Association analysis

REGENIE (V.1.0.6.7)^27^ was used for association testing and accounts for relatedness, among other factors. The null model for association testing was constructed using array genotypes with the following criteria: MAF ≥0.01, maximum Hardy-Weinberg Equilibrium p value of 1e^−15^, genotyping rate of 0.01 and missingness of 0.1 within individuals of European ancestry. We additionally pruned these SNPs based on the linkage structure within a white British subset of the UKB, with a maximum r^2^ of 0.9, and then further only included variants with an MAC of 100. All continuous traits were single inverse normalised prior to regression testing, but all reported means were calculated from non-normalised data. Covariates included were age, sex and centre for all traits. The Strengthening the Reporting of Genetic Association Studies guidelines were used.^28^

#### Copy-number variants

17q12 microdeletions and microduplications were each coded as pseudo-heterozygous SNPs in pedigree format (PED) and the meta-files required for the association testing against the null model were generated using Plink (V.2.00a2LM).^29^

#### 17q12 single-nucleotide polymorphism

**Figure 1:**
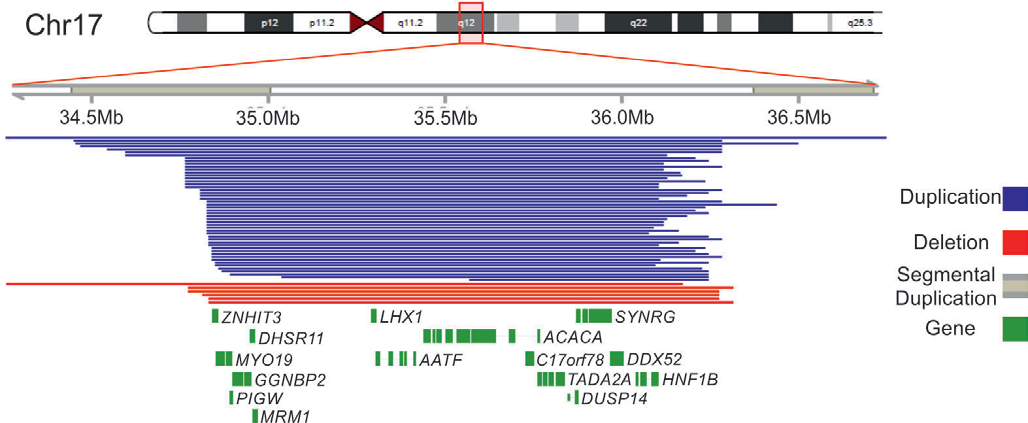
17q12 genomic region highlighting segmental microduplications, microdeletions and microduplications identified in this study. Only the unique detectable regions for microdeletions (n=6) and microduplications (n=50), based on CNV calling, are shown. The remaining identified microdeletions (n=5) and microduplications (n=56) share breakpoints, with one which is plotted.

SNPs in the 17q12 region were tested against all phenotypes for associations. There were obvious areas where SNPs were absent from this region which correlated with the known segmental microduplications (figure 1). We applied a significance threshold of p<9×10^−5^ (ie, p=0.05/535) because we tested 535 SNPs in the region.

#### Exome variant burden testing

REGENIE-GENE^27^ was used for burden testing of each of the 15 genes in the 17q12 region against all phenotypes. Burden tests were carried out for PTVs with and without pathogenic missense variants as well as single-gene burdens. No participants with microdeletions also had a PTV in any of the other genes. We applied a significance threshold of p<3×10^−3^ (ie, p=0.05/15 genes tested).

## Results

### Eleven 17q12 microdeletion and 106 17q12 microduplication carriers were identified in UKB

Using SNP array data, we found 11 microdeletions and 106 microduplications in the UKB cohort, ranging in size between 1.4–7.0 Mb and 1.2–7.0 Mb, respectively (figure 1). Ten microdeletions and 100 microduplications were identified in individuals of European ancestry and were included in downstream association analyses. Cohort clinical characteristics are included in online supplemental table 2.

### Recurrent 17q12 microdeletions and microduplications are both associated with renal disease

eGFR (mL/min/1.73 m^2^) was consistently lower in individuals with microdeletions (n=9, mean=63.95; ß=−1.91, 95% CI −2.45 to –1.37, p=3×10^−12^) and microduplications (n=94, mean=77.66; ß=−0.87, 95% CI −1.04 to –0.71, p=6×10^−25^), relative to controls (mean=92.72) (table 1 and online supplemental table 2). Those with microduplications also had significantly higher eGFR than those with microdeletions (ß=0.63, 95% CI 0.47 to 0.79, p<7×10^−15^).

**Table 1:** Genotype–phenotype association analysis for 17q12 microdeletions (n=10) and microduplications (n=100) in participants of European ancestry in the UK Biobank (continuous traits)

Phenotype	Deletions				Duplications			
	Beta	SE	95% CI	P value	Beta	SE	95% CI	P value
eGFR	−1.91	0.27	−2.18 to −1.64	3×10^−12^	−0.87	0.08	−1.04 to −0.71	6×10^−25^
ACR	0.45	0.30	−0.14 to 1.04	1×10^−1^	0.40	0.10	0.21 to 0.59	4×10^−5^
Urea	1.84	0.31	1.23 to 2.45	3×10^−9^	0.68	0.10	0.49 to 0.87	1×10^−12^
Total protein	−0.27	0.32	−0.90 to 0.35	4×10^−1^	0.36	0.10	0.16 to 0.56	3×10^−4^
HbA1c	1.26	0.28	0.71 to 1.81	7×10^−6^	0.11	0.09	−0.07 to 0.29	3×10^−1^
CRP	−0.47	0.31	−1.08 to 0.14	1×10^−1^	0.44	0.10	0.25 to 0.63	4×10^−6^
ALP	1.52	0.29	0.95 to 2.10	2×10^−7^	0.07	0.09	−0.11 to 0.25	4×10^−1^
AST	0.83	0.31	0.22 to 1.43	7×10^−3^	0.21	0.10	0.03 to 0.40	3×10^−2^
ALT	1.15	0.30	0.55 to 1.74	2×10^−4^	0.03	0.09	−0.16 to 0.21	8×10^−1^
GGT	0.81	0.29	0.24 to 1.38	5×10^−3^	0.09	0.09	−0.09 to 0.26	3×10^−1^
FI	−0.30	0.43	−1.15 to 0.54	5×10^−1^	−0.59	0.16	−0.91 to −0.27	3×10^−4^
Years in education	−0.85	0.30	−1.44 to −0.27	4×10^−3^	−0.32	0.10	−0.51 to −0.13	8×10^−4^
Job class	0.64	0.49	−0.32 to 1.61	2×10^−1^	0.53	0.13	0.28 to 0.79	5×10^−5^
Income	−1.15	0.35	−1.82 to −0.47	9×10^−4^	−0.23	0.10	−0.44 to −0.03	2×10^−2^
TDI	0.73	0.30	0.15 to 1.32	1×10^−2^	0.24	0.10	0.05 to 0.43	9×10^−3^
Qualifications	−0.86	0.30	−1.45 to −0.27	4×10^−3^	−0.29	0.10	−0.48 to −0.11	2×10^−3^

See online supplemental table 1 for phenotype details.

ACR, urinary albumin creatinine ratio; ALP, alkaline phosphatase; ALT, alanine aminotransferase; AST, aspartate aminotransferase; beta, regression coefficient; CRP, C reactive protein; eGFR, estimated glomerular filtration rate (mL/min/1.73 m^2^; Chronic Kidney Disease Epidemiology Collaboration Creatinine-Cystatin 2012); FI, fluid intelligence; GGT, gamma glutamyl transferase; HbA1c, glycated haemoglobin; TDI, Townsend Deprivation Index.

**Figure 2:**
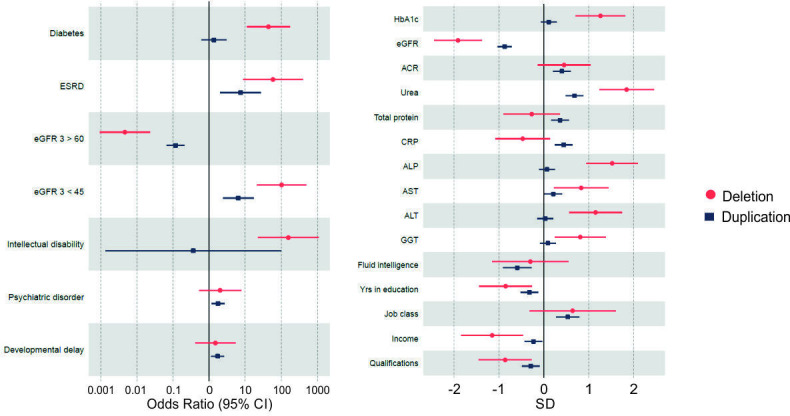
Forest plots of 17q12 microdeletions and microduplication disease associations in the UK Biobank. Left panel: binary traits; right panel: inverse normalised continuous traits. Note that SD below 0 represents negative associations. ACR, urinary albumin to creatinine ratio; ALP, alkaline phosphatase; ALT, alanine aminotransferase; AST, aspartate aminotransferase; CRP, C reactive protein; eGFR, estimated glomerular filtration rate; ESRD, end-stage renal disease; GGT, gamma glutamyl transferase; HbA1c, glycated haemoglobin.

We found 2913 out of 449 651 controls (0.65%) had moderately decreased renal function or ESRD (eGFR <45 plus renal replacement therapies), which was a significantly lower proportion than individuals with either microdeletions (20%; OR=121.96, 95% CI 25.18 to 590.75, p=2×10^−5^) or microduplications (4%; OR=6.25, 95% CI 2.33 to 16.75, p=3×10^−3^) when compared with eGFR ≥45 as the controls. Furthermore, 1339 individuals out of 450 879 controls (0.3%) had ESRD in the UKB (age range 40.3–70.3). Again, this was a significantly lower proportion than observed in individuals with microdeletions (n=1; OR=63.29, 95% CI 8.710 to 459.92, p<0.003, age=43.25) or microduplications (n=2; OR=9.46, 95% CI 2.61 to 34.31, p=0.01, ages=65.5 and 68.1) (table 2 and figure 2).

**Table 2:** Genotype–phenotype association analysis for 17q12 microdeletions (n=10) and microduplications (n=100) in participants of European ancestry in the UK Biobank (binary traits)

Phenotype	Deletions				Duplications			
	OR	SE	95% CI	P value	OR	SE	95% CI	P value
Diabetes	43.74	2.02	11.06 to 172.94	2×10^−7^	1.37	1.49	0.63 to 2.98	4×10^−1^
ESRD	63.29	2.75	8.71 to 459.92	3×10^−3^	9.46	1.93	2.61 to 34.31	9×10^−3^
eGFR <60	213.9	2.24	44.12 to 1036.96	2×10^−9^	8.47	1.34	4.76 to 15.05	1×10^−9^
eGFR <45	121.96	2.24	25.18 to 590.75	2×10^−5^	6.24	1.65	2.33 to 16.75	3×10^−3^
ID	155.94	2.69	22.37 to 1087.02	6×10^−4^	0.37	17.23	1×10^−3^ to 96.92	7×10^−1^
Psych	2.01	1.97	0.53 to 7.62	3×10^−1^	1.75	1.23	1.17 to 2.61	7×10^−3^
DD	1.49	1.91	0.42 to 5.32	5×10^−1^	1.73	1.23	1.15 to 2.61	7×10^−3^

See online supplemental table 1 for phenotype details.

DD, developmental delay; eGFR, estimated glomerular filtration rate (mL/min/1.73 m^2^; Chronic Kidney Disease Epidemiology Collaboration Creatinine-Cystatin 2012); ESRD, end-stage renal disease; GP, General Practitioner; HCP, Health Care Professional; ID, diagnosed intellectual disability; Psych, visit GP or HCP for psychiatric disorder.

Individuals with microdeletions had lower urinary ACR (mg/mmol) than those with microduplications (ß=−0.32, 95% CI −0.50 to –0.14, p=5×10^−4^) when compared directly. When compared with the background population (mean=1.98), ACR in microduplications was significantly raised (mean=5.41, ß=0.40, 95% CI 0.21 to 0.59, p=4×10^−5^) but not in microdeletions (mean=2.32, ß=0.45, 95% CI −0.14 to 1.03, p=0.13). Three participants in the microdeletion cohort had an ACR >60 mg/mmol, of whom one had ESRD and another had a moderately decreased eGFR of 51 mL/min/1.73 m^2^.

### Recurrent 17q12 microdeletions cause diabetes and abnormal liver function tests, but microduplications do not

As has been published previously, we found that 17q12 microdeletions were strongly associated with diabetes (OR=43.74, 95% CI 11.06 to 172.94, p=2×10^−7^), whereas the reciprocal microduplications were not (OR=1.37, 95% CI 0.63 to 2.98, p=0.4).

Similarly, microdeletions were associated with raised liver enzymes: ALP (U/L) (mean=196.9, ß=1.52, 95% CI 0.95 to 2.1, p=2×10^−7^), ALT (U/L) (mean=49.24, ß=1.15, 95% CI 0.55 to 1.74, p=2×10^−4^), AST (U/L) (mean=65.74, ß=0.83, 95% CI 0.22 to 1.43, p=7×10^−3^) and GGT (U/L) (mean=112.0, ß=0.81, 95% CI 0.24 to 1.38, p=5×10^−3^). Direct bilirubin and total bilirubin were not significantly different in either microdeletions (ß=0.26, 95% CI −0.43 to 0.94, p=0.46; ß=−0.17, 95% CI −0.68 to 0.35, p=0.53) or microduplications (ß=−0.02, 95% CI −0.20 to 0.16, p=0.84; ß=0.05, 95% CI −0.11 to 0.21, p=0.56) (figure 2).

### Cognitive ability is negatively affected in both 17q12 microdeletions and microduplications

Fluid intelligence was significantly lower in 17q12 recurrent microduplications (ß=−0.59, 95% CI −0.91 to –0.27, p=3×10^−4^) but not in the reciprocal microdeletions (ß=−0.30, 95% CI −1.15 to 0.54, p=0.48), although the CIs overlapped. There was also an association with fewer years in education (microdeletions: ß=−0.85, 95% CI −1.44 to –0.27, p=4×10^−3^; microduplications: ß=−0.32, 95% CI −0.51 to –0.13, p=8×10^−4^) as well as lower educational attainment (microdeletions: ß=−0.86, 95% CI −1.45 to –0.27, p=4×10^−3^; microduplications: ß=−0.29, 95% CI −0.48 to –0.11, p=2×10^−3^). Additionally, microdeletions are associated with lower income (ß=−1.15, 95% CI −1.82 to –0.47, p=9×10^−4^) and microduplications associated with lower job class (ß=0.53, 95% CI 0.28 to 0.79, p=5×10^−5^).

Duplications are associated with developmental delay (OR=1.73, 95% CI 1.15 to 2.61, p=2×10^−3^); however, microdeletions are not (OR=1.49, 95% CI 0.42 to 5.32, p=0.54), although the CIs overlapped. Microdeletions are more likely to result in a diagnosed intellectual disability (OR=155.94, 95% CI 22.37 to 1087.02, p=6×10^−4^). Individuals with a microduplication were also more likely to visit either a general practitioner or a psychiatrist for psychiatric disorder (OR=1.75, 95% CI 1.17 to 2.61, p=2×10^−3^) (figure 2).

### SNP associations in the 17q12 region suggest that genes other than *HNF1B* may contribute to the observed phenotypes

**Figure 3:**
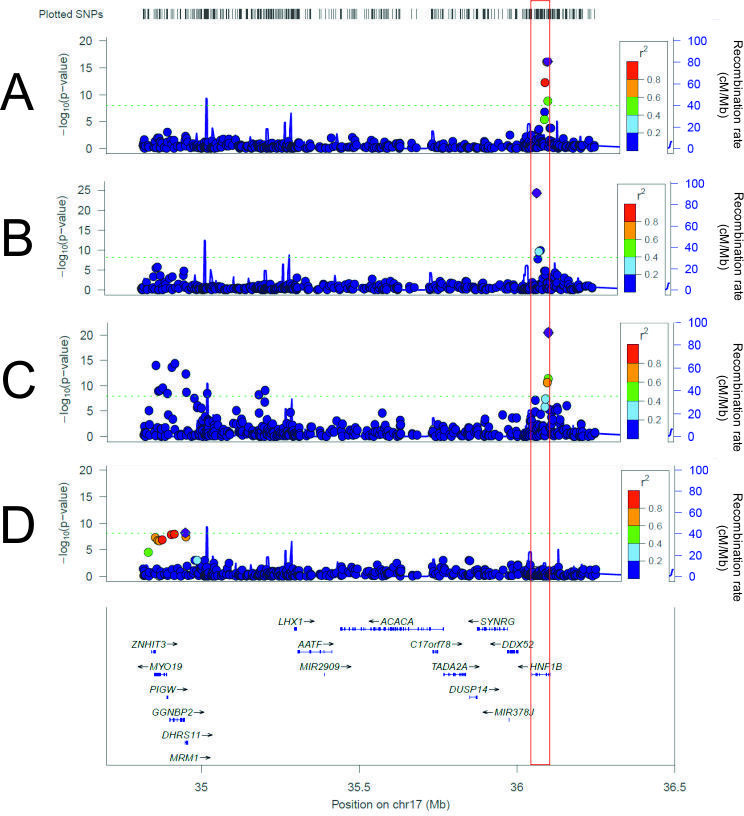
LocusZoom plots of GWAS SNP testing: (A) diabetes, (B) alanine aminotransferase, (C) estimated glomerular filtration rate and (D) fluid intelligence. The red vertical box indicates *HNF1B* location. The green dashed line indicates genome-wide significance at 1×10^−8^.

We identified a genomic risk locus at *HNF1B* for diabetes (NC_000017.11:g.37741165C>G, rs7501939, p=6×10^−17^), increased ALT (NC_000017.11:g.37713312C>T, rs17138478, p=6×10^−25^), AST (NC_000017.11:g.37713312C>T, rs17138478, p=4×10^−12^) and GGT (NC_000017.11:g.37717101A>G, rs718961, p=2×10^−52^). For eGFR, two risk loci were identified: one around *HNF1B* (NC_000017.11:g.37741642T>G, rs12601991, p=4×10^−21^), as expected, and another at the other end of the deletion region (NC_000017.11:g.36558947T>C, rs12150665, p=4×10^−15^), encompassing five nearby genes (figure 3). Furthermore, this second locus was also associated with decreased fluid intelligence (NC_000017.11:g.36593168T>G, rs1005552, p=6×10^−9^), while the *HNF1B* locus was not. This result suggests that, although *HNF1B* is the primary driver of decreased eGFR, it may not be the sole contributor and that a gene or genes in this second locus may explain the neurocognitive phenotypes (figure 3).

Colocalisation analysis between eGFR and fluid intelligence for all the SNPs in the region (n=535) predicted that both traits associate with the region but with different causal variants (posterior probability=0.99). When testing each genomic risk locus separately, the 5’ locus (n=199 SNPs) predicted that both traits are associated and share a single causal variant (posterior probability=0.995), whereas the 3’ locus (n=336) indicated that only eGFR was associated with the region (posterior probability=0.98).

### Burden testing of 17q12 genes does not provide evidence of individual gene contributions to microdeletion phenotypes

Two PTVs and one pathogenic missense variant in *HNF1B* were identified in four UKB participants of European ancestry, one of whom had diabetes (p=0.06). These variants were associated with increased ACR (ß=1.66, 95% CI 0.73 to 2.58, p=4×10^−4^; background: mean=17.53, range=1.32–18 932.28, median=9.80; *HNF1B* pathogenic background: mean=53.83, range=13.09–105.04, median=48.61). We also tested the associations with PTVs and rare missense variants if they had an MAC ≤30, LOFTEE^21^ high-confidence LoF or a CADD score >30^22^ in the 14 other genes in the 17q12 region. None of these variant groups was associated with decreased eGFR. *ZNHIT3* (38 PTVs and 13 missense heterozygotes) was associated with structural renal abnormalities (OR=52.18, 95% CI 7.59 to 358.83, p=4×10^−4^); one individual had a horseshoe kidney (89 in UKB), one had a congenital bile duct malformation (18 in UKB) and one had an unspecified endocrine congenital malformation (121 in UKB). We also found associations between rare PTV/missense variants in *C17orf78* with elevated cystatin C (ß=0.51, 95% CI 0.23 to 0.80, p=3×10^−4^) and *SYNRG* with increased risk of bipolar disorder (OR=27.45, 95% CI 3.03 to 248.61, p=8×10^−4^).

## Discussion

We have shown that the prevalence of 17q12 recurrent microduplications is 1 in 4607 (n=106) and 1 in 44 398 for microdeletions (n=11) in a population-based cohort of 488 377 individuals in the UKB. We have confirmed that microdeletions associate with both diabetes and raised liver enzymes, whereas microduplications do not, consistent with previous publications in smaller cohorts.^12 13 30–32^

Both microdeletions and microduplications are associated with decreased eGFR, despite only a few individuals reaching ESRD (n=1 and n=2, respectively). Microdeletions show a greater effect size than microduplications, which is consistent with other shared phenotypes.^3 6^ Heterozygous variants in *HNF1B* are one of the most common monogenic causes of developmental kidney disease.^33^ Rare cases of renal malformations have been reported in microduplication carriers,^34^ but systematic imaging of affected individuals has not been published so far, to the best of our knowledge. One of the limitations of this work was the small number of MRI results available for UKB participants, particularly for a condition like *HNF1B*-associated disease, where structural abnormalities are a key phenotypic feature. 17q12 microdeletions are known to cause diabetes and renal disease.^1^ In this cohort we identify one individual with a microdeletion who has diabetes and ESRD, and five individuals with microdeletions who have diabetes and no ESRD. ESRD is a life-threatening disease status and represents severe progression of the observed 17q12 deletion syndrome. It is therefore not surprising that not all participants have late-stage disease progression, such as ESRD, particularly in a population cohort where participants would typically be assumed to be in ‘good health’ to be able to participate. We found an association with 17q12 microduplications and ESRD, but not diabetes. Diabetes can itself cause ESRD and we identify one individual with a microduplication who has diabetes and ESRD and one individual with a microduplication who has ESRD without diabetes. It does remain a possibility that the ESRD observed in the individuals with a microduplication could be caused by their diabetes. Given the low numbers of affected individuals, untangling the intricacies of these potential comorbidities and the underlying genetic drivers would be a suitable topic for further research.

Microdeletions and microduplications cause neurodevelopmental disorders. Microduplications are also associated with psychiatric disorders.^1 4^ We show intelligence traits are decreased in individuals with both microdeletions and microduplications, with microdeletions being more severe. Microdeletions were not associated with decreased fluid intelligence, but this likely reflects low statistical power. One individual with a microdeletion had mental retardation (ICD10: F70–79). This was one of only 144 participants in the UKB cohort reported to have this diagnosis. Microduplications were associated with developmental delay with a similar effect size to microdeletions. Microduplications were associated with visiting a healthcare professional for a psychiatric disorder, but not for individual psychiatric traits. This is likely due to the challenge of diagnosing neurodevelopmental and psychiatric conditions and the low incidence in the UKB due to recruitment bias. We found a genomic risk locus for fluid intelligence which was distant from *HNF1B*, reaffirming previous findings that other genes are likely responsible for neurodevelopmental phenotypes in 17q12 microdeletion and microduplication carriers.^14 35^

Intragenic *HNF1B* pathogenic variants cause lower eGFR than 17q12 microdeletions, suggesting a dominant negative effect of the former.^36^ Association testing of common SNPs in the region identified a second genomic risk locus for eGFR, with an intronic SNP in *GGNBP2* (NC_000017.11:g.36558947T>C, rs12150665) as the most significant. Gene burden testing associated *ZNHIT3* with an increased likelihood of structural abnormalities, but also *C17orf78* with increased cystatin C. Overexpression of *ACACA* causes podocyte cell death in vitro and may therefore cause the decreased eGFR in microduplications. This mechanism would also support the association with microduplications and raised ACR. *ACACA* knockdown does not exhibit a significant protective effect.^37^ Association and colocalisation analyses strongly suggest that there are two independent genetic effects across the 17q12 variable-copy region and that *HNF1B* is unlikely the sole contributor to renal phenotypes in both microdeletions and microduplications.

To date, 17q12 CNVs have largely been studied in selective cohorts, which has led to ascertainment bias. The UKB is a valuable resource that can be leveraged to discover genotype–phenotype associations but is enriched in participants of higher socioeconomic stratification and lower rates of disease. This means studying rare genetic variation underlying complex, and often severe, disease presentation is more challenging. Specifically, the rarer the genetic variation and the more severe the phenotype, the less likely it is to be observed in a population cohort. Similarly, some phenotypes are more easily captured and documented in a population cohort than others. Examples in this study include psychiatric conditions but also the lack of MRI in affected individuals. The latter is a drawback in a condition like *HNF1B*-associated disease, where structural abnormalities are one of the main phenotypic features. It can also be challenging to identify the age of onset of disorders and whether they occur in parallel or isolation. Despite this, carriers of 17q12 microdeletions and microduplications are of a similar age at recruitment to the general population (online supplemental table 2).

## Conclusions

We show that both 17q12 microdeletions and microduplications are associated with renal disease and provide evidence that *HNF1B* is unlikely to be the sole contributor to all associated phenotypes. This work highlights the utility of population cohorts with high-resolution genomic and phenotypic data, such as UKB, to study multigene disorders. As population data sets increase in size and the accuracy of variant detection and interpretation improves, we will be better able to characterise the genotype–phenotype associations in complex multisystem disorders like 17q12 deletion and microduplications.

## Data Availability

All data relevant to the study are included in the article or uploaded as supplementary information.
