# Supplementary material for: Recurrent 17q12 microduplications contribute to renal disease but not diabetes
Source: J Med Genet. 2022 Sep 15;60(5):491–7. doi: 10.1136/jmg-2022-108615 (PMC10176419; doi:10.1136/jmg-2022-108615)
Supplement: Supplementary data [file jmg-2022-108615supp001.pdf]

Phenotype	ICD10/UKB code	Details
Renal disease (ESRD or eGFR <15)	N180	End stage renal disease
	N185	CKD stage 5
	T861	Complications of kidney transplant
	Z940	Kidney transplant status
	Z992	Dependence on renal dialysis
	Z492	Other dialysis
	Z491	Extracorporeal dialysis
	Z490	Preparatory care for dialysis
	T861	Complications of kidney transplant
Other kidney malformations	N27	Small kidney
	Q63	Other congenital malformations of kidney
Malformations of ureter	Q621	Congenital occlusion of ureter
	Q622	Congenital mega ureter
	Q623	Other obstructive defects of renal pelvis and ureter
	Q624	Agenesis of ureter
	Q625	Duplication of ureter
	Q626	Malposition of ureter
	Q627	Congenital vesico-uretero-renal reflux
	Q628	Other congenital malformations of ureter
Kidney Cysts	N281	Cyst of kidney, acquired
	Q612	Polycystic kidney, adult type
Structural malformations of the kidney	Q60	Renal agenesis and other reduction defects of kidney
	Q61	Cystic kidney disease
	Q620	Congenital hydronephrosis
	Q631	Lobulated, fused and horseshoe kidney
Family history of renal disease	Z841	Family history of disorders of kidney and ureter
Disorders of kidney or ureter	N288	Other specified disorders of kidney and ureter
	N289	Disorder of kidney and ureter, unspecified

eGFR	30700	Creatinine
	30720	Cystatin C
ACR	30500	Microalbumin in urine
	30510	Creatinine (enzymatic) in urine
Diabetes	30750	Glycated haemoglobin (HbA1c)
	20003	Medications - insulin, sulfonylureas, metformin
Years in education	22501	Years in education
Fluid intelligence	20016	Fluid intelligence
Income	26411	Income
Job class	20277	Job class
Qualifications	6138	Qualifications
Urinary biomarkers	30500	Microalbumin in urine
	30510	Creatinine (enzymatic) in urine
	30520	Potassium in urine
	30530	Sodium in urine
Serum biomarkers	30600	Albumin
	30610	Alkaline phosphatase
	30620	Alanine aminotransferase
	30630	Apolipoprotein A
	30640	Apolipoprotein B
	30650	Aspartate aminotransferase
	30660	Direct bilirubin
	30670	Urea
	30680	Calcium
	30690	Cholesterol
	30700	Creatinine
	30710	C-reactive protein
	30720	Cystatin C
	30730	Gamma glutamyltransferase

	30740	Glucose
	30750	Glycated haemoglobin (HbA1c)
	30760	HDL cholesterol
	30770	IGF-1
	30780	LDL direct
	30790	Lipoprotein A
	30800	Oestradiol
	30810	Phosphate
	30820	Rheumatoid factor
	30830	SHBG
	30840	Total bilirubin
	30850	Testosterone
	30860	Total protein
	30870	Triglycerides
	30880	Urate
	30890	Vitamin D
Poor mental state	2030	Guilty feelings
	1950	Sensitivity/ hurt feelins
	1940	irritability
	2020	Loneliness, isolation
	1930	Miserableness
	1920	Mood swings
	1970	Nervous feelings
	2040	Risk taking
	2010	Suffer from 'nerves'
	1990	Tense/'highly strung'
	1980	Worrier/anxious feelings
	2000	Worry too long after embarrassment
Intellectual disability	F70	Mild intellectual disabilities
	F71	Moderate intellectual disabilities

	F72	Severe intellectual disabilities
	F73	Profound intellectual disabilities
	F78	Other intellectual disabilities
	F79	Unspecified intellectual disabilities
	F800	Phonological disorder
	F801	Expressive language disorder
	F802	Mixed receptive-expressive language disorder
	F804	Speech and language developmental delay due to hearing loss
	F808	Other developmental disorders of speech and language
	F809	Developmental disorders of speech and language, unspecified
	F810	Specific reading disorder
	F812	Mathematics disorder
	F818	Other developmental disorders of scholastic skills
	F819	Developmental disorders of scholastic skills, unspecified
	F82	Specific developmental disorder of motor function
Visited healthcare professional for psychiatric disorder	2090	Seen GP for nerves, anxiety, tension or depression
	2100	Seen a psychiatrist for nerves, anxiety, tension or depression
Epilepsy	131048	Date G40 first reported (epilepsy)
	G400	Localisation related idiopathic epilepsy and epileptic syndromes with seizures of localised onset
	G401	Localisation related idiopathic epilepsy and epileptic syndromes with simple partial seizures
	G402	Localisation related idiopathic epilepsy and epileptic syndromes with complex partial seizures
	G403	Generalised idiopathic epilepsy and epileptic syndromes
	G404	Other generalised epilepsy and epileptic syndromes
	G408	Other epilepsy and recurrent seizures
	G409	Epilepsy, unspecified
	G40A	Absence epileptic syndrome

Schizophrenia	F20	Schizophrenia
	F21	schizotypal disorder
	F22	Delusional disorders
	F23	Brief psychotic disorder
	F24	Shared psychotic disorder
	F25	Schizoaffective disorders
	F28	Other psychotic disorder not due to a substance or known physiological condition
	F29	Unspecified psychosis not due to a substance or known physiological condition
Bipolar	F30	Manic episode
	F31	Bipolar disorder
	F32	Major depressive disorder, single episode
	F33	Major depressive disorder, recurrent
	F34	Persistent mood [affective] disorders
	F38	Other mood affective disorder
	F39	Unspecified mood affective disorder
	20122	Bipolar disorder status
Congenital malformation	Q0	Nervous system
	Q1	Eye, ear, face, neck
	Q2	Circulatory system
	Q3	Respiratory system, cleft lip and palate
	Q4	Digestive system
	Q5	Genital organs
	Q6	Urinary system
	Q7	Musculoskeletal system
	Q8	Other
	Q9	Chromosomal abnormalities, not elsewhere classified
Pervasive	F840	Autistic disorder
	F842	Rett’s syndrome
	F843	Other childhood disintegrative disorder

	F845	Asperger's syndrome
	F848	Other pervasive developmental disorders
	F849	Pervasive developmental disorder, unspecified
Developmental delay	As above	Bipolar
	As above	Schizophrenia
	As above	Pervasive
	As above	Visited healthcare professional for psychiatric disorder
	As above	Intellectual disability
	As above	Malformation
	As above	Epilepsy

Supplementary table 1 – Phenotype definitions

Category	UKB background					17q12 Microdeletion					17q12 Microduplication				
	Mean	95%CI	n	%	Total	Mean	95%CI	n	%	Total	Mean	95%CI	n	%	Total
**Participants**	-	-	450,879	89.75	502,371	-	-	10	90.91	11	-	-	100	94.34	106
**Sex (male)**	-	-	206,140	45.72	450,879	-	-	5	50	10	-	-	6	60	100
**Age (years)**	57.28	57.26, 57.31	-	-	450,879	52.58	46.22-58.93	-	-	10	56.84	55.19-58.48	-	-	100
**BMI**	27.40	27.38-27.41	-	-	449,140	24.87	22.86-26.88	-	-	10	28.35	27.27-29.43	-	-	100
**Diabetes**	-	-	30,191	6.70	450,879	-	-	6	60	10	-	-	9	9	100
**eGFR**	92.72	92.67-92.77	-	-	429,395	63.95	50.59-77.32	-	-	9	77.66	73.96-81.37	-	-	94
**ACR**	1.98	1.95-2.01	-	-	437,935	2.32	0.62-4.03	-	-	10	5.41	1.67-9.15	-	-	95
**ALP**	83.59	83.52-83.67	-	-	429,948	196.9	33.63-360.24	-	-	9	85.13	80.06-90.21	-	-	94
**AST**	26.21	26.18-26.24	-	-	428,348	65.74	-12.62-144.11	-	-	9	27.50	26.06-28.93	-	-	94
**ALT**	23.54	23.94-23.58	-	-	429,777	49.24	12.42-86.07	-	-	9	24.79	22.25-27.33	-	-	94
**GGT**	37.37	37.24-37.50	-	-	429,706	112.0	10.33-213.72	-	-	9	40.82	34.78-46.86	-	-	94
**Smoking**	-	-	202,204	45.46	444,793	-	-	4	40	10	-	-	50	51.02	98
**Obesity**	-	-	110,326	24.47	450,879	-	-	0	0	10	-	-	30	30	100
**CKD Family history**	-	-	61	0.01	450,879	-	-	0	0	10	-	-	0	0	100
**Hypertension**	-	-	241,572	53.92	448,000	-	-	3	30	10	-	-	59	59	100
**BP medication**	-	-	92,981	20.77	447,703	-	-	3	30	10	-	-	29	29	100
**Systolic BP**	144.24	144.17-144.31	-	-	449,857	136.6	118.94-154.26	-	-	10	146.36	141.24-151.48	-	-	100
**Diastolic BP**	86.35	86.64-86.69	-	-	449,857	88.25	77.83-98.37	-	-	10	87.52	84.69-90.35	-	-	100
**ESRD**	-	-	1,339	0.30	450,879	-	-	1	10	10	-	-	2	2	100

Supplementary Table 2 – Cohort clinical characteristics. UKB background = EU ancestry excluding; 17q12 microdeletions, microduplications and *HNF1B* pathogenic mutations. Age = age at recruitment to UKB study. Smoking = if participants had ever smoked. Obesity calculated from BMI. Hypertension based on blood pressure medications and blood pressure. ESRD = eGFR < 15 plus participants with renal replacement therapies; ALP = alkaline phosphatase; AST = aspartate amino transferase; ALT = alanine aminotransferase; GGT = gamma-glutamyl transferase;
